# Treatment of right ventricular perforation during percutaneous coronary intervention

**DOI:** 10.5830/CVJA-2014-072

**Published:** 2015

**Authors:** Guoqiang Gu, Jidong Zhang, We Cui

**Affiliations:** Department of Cardiology, Hebei Institute of Cardiology, Second Hospital of Hebei Medical University, Shijiazhuang, Hebei, China; Department of Cardiology, Hebei Institute of Cardiology, Second Hospital of Hebei Medical University, Shijiazhuang, Hebei, China; Department of Cardiology, Hebei Institute of Cardiology, Second Hospital of Hebei Medical University, Shijiazhuang, Hebei, China

**Keywords:** percutaneous coronary intervention (PCI), coronary artery perforation, myocardial cell necrosis, right ventricle, cardiac tamponade

## Abstract

Percutaneous coronary intervention (PCI) is widely used to treat stenotic coronary arteries caused by coronary heart disease. Coronary artery perforation is a rare but dreaded complication of PCI. Here, we report the successful treatment of a patient with coronary perforation of the right ventricular cavity. To our knowledge, this is the first report of its kind.

The patient was a 69-year-old woman with intermittent chest tightness and chest pain of about five years’ duration who was hospitalised for severe chest tightness and pain persisting for three days. She had a history of hypertension and hyperlipidaemia; routine admission examination showed no other abnormalities. Results of routine blood, urine and stool tests, liver and kidney function, clotting time, electrocardiogram, chest radiography and echocardiography were normal.

Although coil embolisation rather than balloon is safe and effective for treating coronary artery perforation, it may be not the best choice overall. If the perforation breaks through into the right ventricle, we may just monitor closely rather than treat. That course may be beneficial for patients in that it reduces the risk of myocardial cell necrosis. This case provides useful information for the treatment of such patients in the future.

## Abstract

Percutaneous coronary intervention (PCI) is a widely used non-surgical procedure to treat stenotic coronary arteries caused by coronary heart disease.^1^,^2^ The benefit of PCI to the patient is great, but the procedure is accompanied by risk. Coronary artery perforation is a rare but dreaded complication of PCI, with a reported incidence from 0.12–0.93% and a mortality rate of about 7–41%.^3^–^14^

In most cases, the perforation breaks through into the pericardium, which may cause cardiac tamponade.^15^ Coronary perforation can also involve the cardiac chambers.^16^ Here we report the successful treatment of a patient with coronary perforation of the right ventricular cavity and provide a brief review of the literature on the treatment of coronary perforation during PCI.

## Case report

The patient was a 69-year-old woman with intermittent chest tightness and chest pain over the previous five years. She was hospitalised for severe chest tightness and chest pain persisting for three days. She had a history of hypertension and hyperlipidaemia; the admission examination showed no other abnormalities. Routine blood, urine and stool tests, liver and kidney function, clotting time, electrocardiogram, chest radiography and echocardiography were normal. A diagnosis of coronary artery disease was considered.

**Figure 1.**
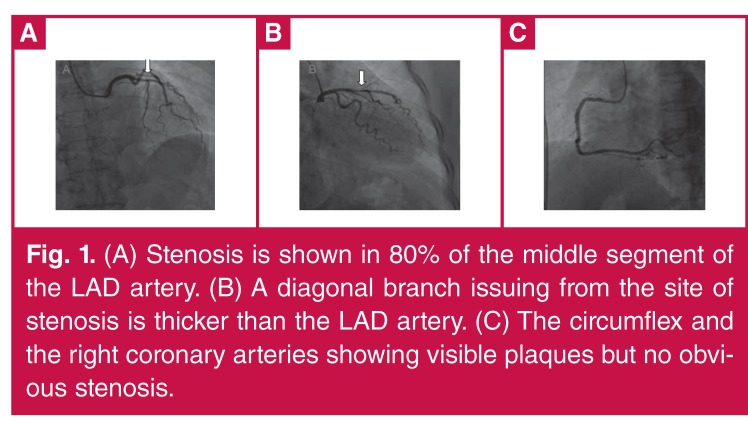
(A) Stenosis is shown in 80% of the middle segment of the LAD artery. (B) A diagonal branch issuing from the site of stenosis is thicker than the LAD artery. (C) The circumflex and the right coronary arteries showing visible plaques but no obvious stenosis.

Coronary angiography showed a right coronary arterydominant circulation. The left main coronary artery was normal, 80% of the middle segment of the left anterior descending (LAD) coronary artery showed stenosis, and the diagonal branch issuing from the site of the stenosis was thicker than the LAD artery. Plaques, but no obvious stenosis, were found in the circumflex and right coronary arteries (Fig. 1A-C).

**Figure 2.**
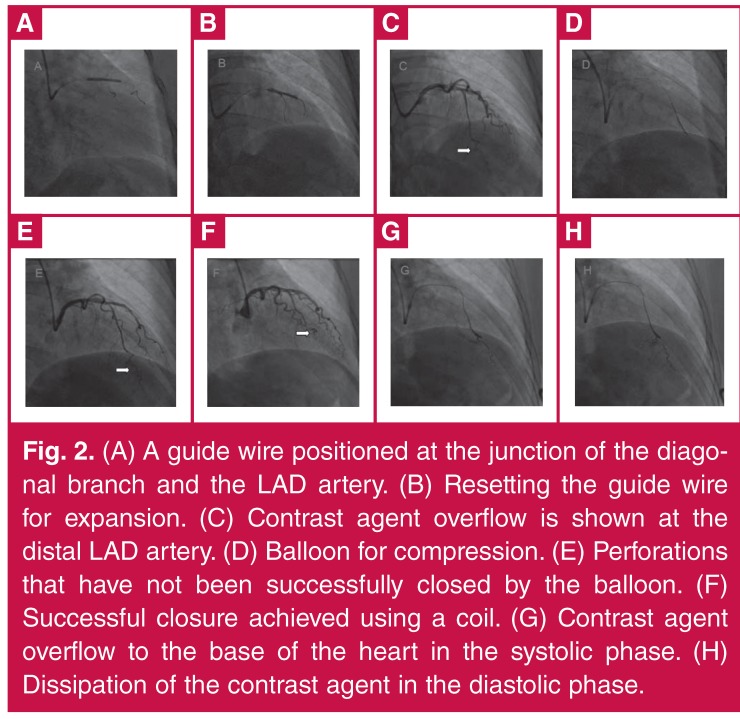
(A) Stenosis is shown in 80% of the middle segment of the LAD artery. (B) A diagonal branch issuing from the site of stenosis is thicker than the LAD artery. (C) The circumflex and the right coronary arteries showing visible plaques but no obvious stenosis.

After discussing treatment with the patient, it was decided to perform PCI of the LAD artery. Because of the narrow opening of the diagonal branch, and because the diagonal branch was thicker than the LAD artery, we planned to implant a stent at the juncture of the diagonal branch and the LAD artery, and to position a guide wire in the artery to protect it (Fig. 2A).

After stent implantation, the guide wire was set for expansion (Fig. 2B). After expansion, angiography showed no mezzanine, side branch occlusion or residual stenosis at the implantation site, and forward blood flow was TIMI grade 3. However, contrast agent overflow was seen at the distal left LAD artery (Fig. 2C). The patient did not experience discomfort and had normal blood pressure with a steady heart rate. As the guide wire did not reach the distal vessel through the perforation site even after several attempts, it was positioned proximal to the perforation site, and a balloon was used for compression (Fig. 2D). Because this did not successfully close the perforation (Fig. 2E), a coil was used to achieve successful closure (Fig. 2F), after obtaining the consent of family members.

## Discussion

The incidence of coronary perforation during PCI is low, but it has a relatively high mortality rate. The available data show that the female gender, increasing age, treatment of a chronic total occlusion, angiographic evidence of calcification, and use of a cutting balloon or rotational atherectomy are associated with increased risk of coronary perforation.^3^–^14^

In a randomly assigned case–control study conducted between 2001 and 2008, Shimony and colleagues found that the strongest predictor of coronary perforation was treatment of a chronic total occlusion.^9^ Gruberg *et al*. identified age and cardiac tamponade as predictors of mortality among patients with coronary perforation.^11^

A classification scheme has been developed to help in the management of patients with perforation and to assist in delivery of optimal care.^10^ Coronary perforation is divided into three classes based on angiographic appearance: I, extraluminal crater without extravasation; II, pericardial or myocardial blushing; III, perforation ≥ 1 mm in diameter with contrast streaming and cavity spilling, i.e. perforation into an anatomical cavity, chamber, or coronary sinus (Ellis type III CS).

Managing coronary perforation during PCI requires an accurate diagnosis of the type of perforation that has occurred. Adverse clinical outcomes (e.g. death or emergency surgical exploration) are associated with angiographic classification of the perforation, and have been more frequently observed in patients who experienced a class III coronary perforation.^8^–^10^,^14^ The management of coronary perforation often includes heparin reversal, discontinuation of glycoprotein IIb/IIIa inhibitors, platelet transfusion, pericardiocentesis, and emergency cardiac surgery. Additional treatment strategies include prolonged balloon inflation, covered stents, injection of polyvinyl alcohol, coil embolisation, and intracoronary administration of autologous blood.^17^–^21^

This patient had normal blood pressure, a steady heart rate and no manifestations of cardiac tamponade during the three-hour procedure. Although balloon occlusion was used within an hour to apply pressure, rapid outward bleeding continued for more than two hours. Ultrasound monitoring of the pericardial cavity was performed during the entire procedure, and overflow into the pericardial fluid was not observed. Imaging showed that the contrast agent overflow visible at the base of the heart in the systolic phase (Fig. 2G) dissipated quickly during diastole (Fig. 2H). Overall, the evidence indicated that this was an Ellis type III CS coronary perforation that penetrated a ventricular cavity.

**Figure 3.**
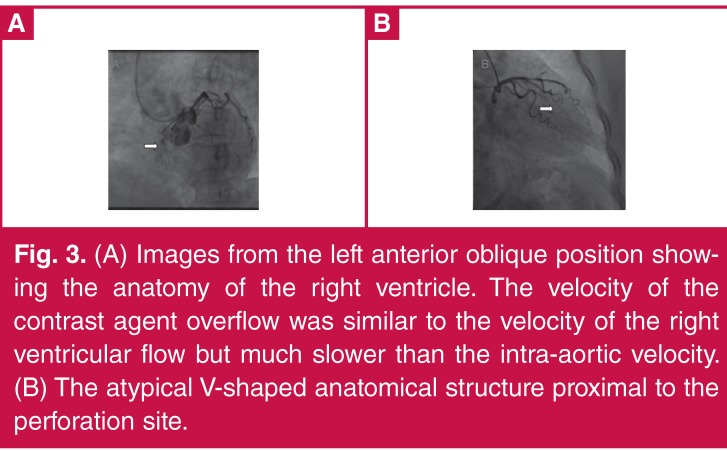
(A) Images from the left anterior oblique position showing the anatomy of the right ventricle. The velocity of the contrast agent overflow was similar to the velocity of the right ventricular flow but much slower than the intra-aortic velocity. (B) The atypical V-shaped anatomical structure proximal to the perforation site.

Evidence for perforation of the right ventricle included the following reasons. First, overflow of contrast agent occurred in both systole and diastole, which is consistent with the haemodynamic properties of the coronary artery and right ventricle. If the left ventricle had been perforated, the contrast agent would have been much more evident in diastole than in systole. Second, images from the left anterior oblique position showing the anatomy of the right ventricle support this interpretation (Fig. 3A). Third, the velocity of the contrast agent overflow was similar to the right ventricular flow velocity, but much slower than the intra-aortic flow velocity (Fig. 3A).

This patient was a 69-year-old woman. The hydrophilic coated guide wire used for expansion and the V-shaped anatomical structure proximal to the perforation site may also have contributed to the perforation (Fig. 3B). Others have found that LAD arteries and tortuous lesions were vulnerable to perforation, and that the guide wire was frequently responsible for the perforation.^8^,^14^ Therefore special care should be exercised to avoid perforation when performing PCI in older females with special anatomical structures.

In the treatment of this patient, balloon compression was unsuccessful. Stent implantation was not considered because the\ vessel lumen was too small. Although it was concluded that the ventricle had been penetrated, for safety reasons, we carried out a block by coil embolisation. We did not use a gelatin sponge because of the risk of pulmonary embolism.

The long- and short-term safety and effectiveness of coil embolisation are good, but it might be not the best choice in all cases. If perforation does involve the right ventricle, close monitoring without any treatment may be beneficial for the patient because of reduction in myocardial cell necrosis.

We did not use glycoprotein IIb/IIIa inhibitors during treatment, therefore the question of discontinuation did not arise. The data show that the more patients who were given a glycoprotein IIb/IIIa inhibitor required the placement of a covered stent or emergency cardiac surgery than those who did not receive it (33.3 vs 3.2%). Clinical outcomes (tamponade, myocardial infarction, death) were similar for patients who had and had not received a glycoprotein IIb/IIIa inhibitor.^8^

Reversal of heparin was considered in our case but was not adopted because of the risk of coronary thrombosis and the patient’s haemodynamic stability. Al-Lamee *et al.* recommend the use of protamine ‘as necessary’ in the setting of coronary perforation if heparin or glycoprotein inhibitors have been administered.^22^

## Conclusion

Coronary artery perforation is a rare but dreaded complication of PCI. Coronary perforation of the right ventricular cavity is less severe than perforation at other sites. Although coil embolisation is a safe and effective alternative to balloon treatment of coronary artery perforation, it might be not the best choice in the short and long term. If the perforation does break through into the right ventricle, we suggest close monitoring rather than treatment, which may be beneficial for patients in that it reduces the risk of myocardial cell necrosis.
